# Quantitative estimates of dietary intake with special emphasis on snacking pattern and nutritional status of free living adults in urban slums of Delhi: impact of nutrition transition

**DOI:** 10.1186/s40795-015-0018-6

**Published:** 2015-10-14

**Authors:** Archna Singh, Vidhu Gupta, Arpita Ghosh, Karen Lock, Suparna Ghosh-Jerath

**Affiliations:** 1 Indian Institute of Public Health—Delhi, Public Health Foundation of India, Plot No. 47, Sector-44, Gurgaon 122002 Haryana, India; 2 All India Institute of Medical Sciences, Delhi, India; 3 Public Health Foundation of India, Haryana, India; 4 London School of Hygiene and Tropical Medicine and Leverhulm Centre for Integrative Research on Agriculture and Health, London, UK

**Keywords:** Overweight, Fat consumption, Snacks, Household dietary diversity, Lipid profile

## Abstract

**Background:**

The nutritional landscape of India is experiencing the fallout of urbanization and globalization. The changes are manifest in dietary patterns as well as health outcomes. The study aimed at assessing household dietary intake pattern with special emphasis on snacking pattern, anthropometric and lipid profiles in low socio-economic status households in an urban slum of Delhi.

**Methods:**

Community based cross-sectional study in 260 households of a purposively selected urban slum in North-East district of Delhi, India. Family dietary surveys including consumption pattern of commercial food products rich in Partially Hydrogenated Vegetable Oils (PHVOs), 24 h dietary recall and assessment of dietary diversity using Household Diet Diversity Scores (HDDS) were done. Assessment of nutritional status using anthropometric and lipid profile on a subsample (*n* =130) were also conducted.

**Results:**

Median energy and fat intake were adequate. Micronutrient intake was found to be inadequate for vitamin A, riboflavin, calcium and folate. *PHVO* usage was low (<20 % households). Milk (39 %), green leafy vegetables (25 %) and fruits (25 %) intake were below recommendations. Mean HDDS was 7.87. Prevalence of overweight/obesity was high (66.7 %). Lipid profile showed mean HDL-C levels lower than recommendations for females.

**Conclusion:**

Community based awareness programs for prevention of non-communicable diseases should incorporate healthy diet and lifestyle practices with emphasis on quantity and quality of nutrient intake. This must be considered as an integral part of chronic disease prevention strategy for underprivileged communities in urban India.

## Background

The rapid economic growth in India has resulted in significant socioeconomic, demographic, nutrition and health transitions within the country [1]. A double burden of malnutrition is being observed due to the coexistence of chronic energy deficiency and overweight in the population driven by influences of urbanization [2]. Further, migration to cities and a disproportionate expansion of settlements within urban slums has resulted in an unhealthy lifestyle because of changes in traditional eating habits, decreased physical activity, exposure to stress, etc. [3, 4]. This is reflected in the increase in over nutrition from 11 % in 1998–99 to 15 % in 2005–06 in NFHS-3. The national data shows a higher prevalence of over-weight/ obesity in women (13 %) as compared to men (9 %). The states of Punjab (30 %), Kerala (28 %) and Delhi (26 %) have the highest prevalence of overweight/obesity in women [5]. Further, overweight and obesity raise the risk of non- communicable diseases (NCDs) [6].

Replacement of traditional diets with energy-dense foods of low nutritional quality [1, 7, 8] is reflected in the increase in the consumption of sugar, oil, milk and animal products [1]. Moreover, the sales of pre-packaged, processed and ready-to-eat meals have been increasing, and projections are that this demand will continue to increase [9, 10]. These foods are usually prepared in Partially Hydrogenated Vegetables Oils (PHVOs) that are the predominant sources of trans fats in Indian diets [2, 11].

In addition to this transition in intake patterns, a suboptimal dietary diversity (which is a qualitative measure of food consumption reflecting household access to a variety of foods) is often observed. In fact, this is frequently a problem among socioeconomically challenged populations from the developing world because their diets are predominantly based on starchy staples with little or no animal products and limited amounts of fresh fruits and vegetables [12]. Hence, communities that are economically or socially underprivileged may be disproportionately vulnerable to the effects of unhealthy diets because of lower levels of awareness, greater economic constraints and limited accessibility to healthier choices. Improving dietary intakes is an important modifiable risk factor for cardiovascular disease (CVD), currently one of the largest contributors to chronic disease burden in India [13]. Increasing fresh fruit and vegetable intake and decreasing fat intake has been recommended as a strategy to improve population health [14]. Further, national and international agencies recommend a modification in the proportion of different types of fat, e.g. removal of trans fat, and a reduction in saturated fat and total energy obtained (en%) from fat in the diet [15].

In India, the recent National Nutrition Monitoring Bureau (NNMB) and National Sample Survey Organization (NSSO) surveys indicate an increase in fat intakes from *PHVOs*—oil [16, 17] with the national average for fat intake reported as 47.9 g per capita per day in urban areas in 2009–10 compared to 42.0 g in 1993–94. High fat consumption is also reported in the urban areas in the states of Punjab (68.9 g per capita per day), Haryana (63.8 g per capita per day) and Gujarat (68.5 g per capita per day).

Despite the availability of data from the national sample surveys, detailed data on population consumption patterns are inaccurate because existing nationally representative surveys are not designed to comprehensively capture intakes from all kinds of foods. Specifically, these surveys do not focus on foods sold in the market that may be prepared in less than optimal quality oils, e.g. those that are high in saturated/trans fats. The inaccuracy in assessment of these fats in the diet is augmented due to multiple food derived sources of unhealthy fats like processed foods, fried foods, etc. and a lack of standardized food preparation methodologies for many ready-to-eat items that are available.

Therefore, the present study was conducted to investigate household dietary intake patterns in a low socioeconomic status (SES) community residing in an urban slum of Delhi. This assessment aimed to capture information on dietary diversity, quality of fat intake, and intake patterns of commercially prepared (fresh and packaged) snacks known to contain high amounts of PHVOs. In addition, the nutritional status of a subset of the study population was assessed by anthropometry and biochemical lipid profile analysis.

## Methods

### Study design

This was a community based cross-sectional study.

### Locale

The study was conducted in a purposively selected, large urban slum: Chanderpuri, in Gandhinagar assembly of North-East district of Delhi. We wanted to capture a typical dietary consumption pattern in an urban poor settlement. The present slum was chosen because it represented an indicative food environment of such settlements. Further, it was a large settlement and hence facilitated better sampling plan with rigour.

In order to estimate the consumption level of trans fats in the study population, a sample size was calculated based on consumption level of 2.5 % of total calories from trans fat, a precision of 0.02, an alpha of 5 % and a non-response rate of 10 %. The final sample size arrived at was 260 households (HHs).

### Sampling framework

Mapping of the selected study area was done by the core project team (comprising the lead researcher and nutritionists) and cluster level consent was taken from the cluster guardian (local political leader). The unit of sampling was the household. After estimating the total number of households at the site, every third household was approached and invited to participate in the study. In order to sample locally available snacks for assessment of their nutrient content, all the vendors operating in the study area were mapped.

The respondents were mostly adult female members of the households (≥18 years of age, preferably married) who were involved in cooking/purchasing of the food. The selection of participants was based on who was primarily in-charge of the kitchen duties. In most households, these were married women present in the HHs. However, HHs where young unmarried women were in-charge of the kitchen, they were selected as the participant/respondent. This criterion of selection of the respondent was based on the social norms in the Indian context [18]. In order to assess nutritional status through assessment of serum lipid profile and anthropometry (see below), an adult active member (a physically fit person, not restricted in their routine activities due to some illness, chronic or otherwise) between 25–60 years of age, belonging to the participating household and consenting to provide a blood sample, was included. Local vendors in the area were contacted for the sampling and purchase of commonly sold, freshly-prepared snacks.

### Time period

The data collection was done between February and March, 2013.

### Recruitment summary

**Fig. 1:**
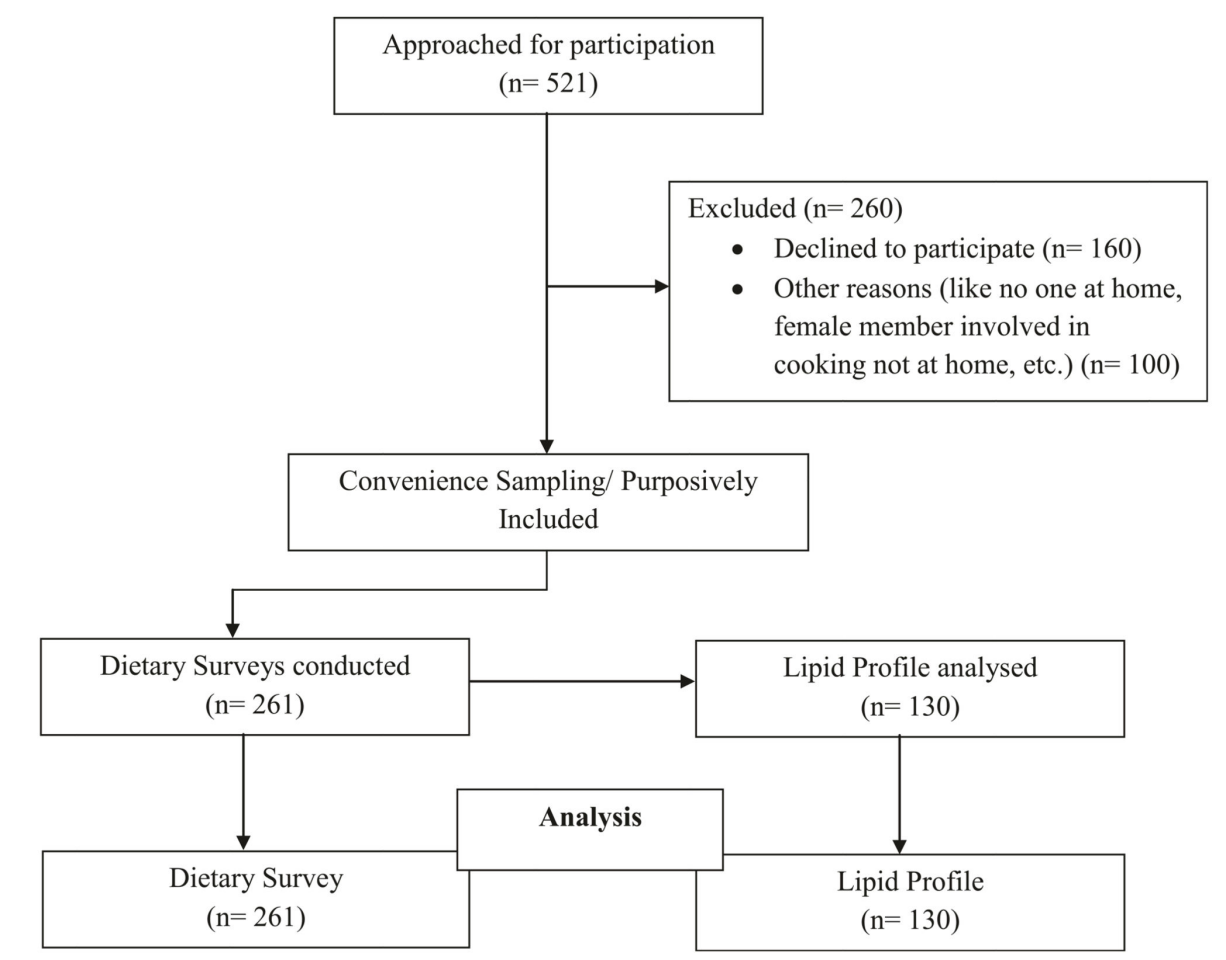
Recruitment Summary of the study

A simple flow chart showing the recruitment of the study population was shown in Fig. 1.

#### Study tools

A pre-tested interviewer administered questionnaire was used for the household survey. The questionnaire elicited and documented information on socio- economic, demographic profile, dietary and snacking patterns at the household level. The snacking pattern questions focused on the kind of snacks consumed in- between meals, meal consumption patterns of working members at their workplace and kind of snacks consumed at workplace or while eating out. Other information included expenditure on purchase of food and snacks as well as the types with amount of oil/fat used for cooking. Apart from the information on food consumption pattern of adult members, questions pertaining to food consumption pattern including snacking pattern of children were also asked. In addition, a household level 24-h dietary recall for two consecutive days was conducted. This method has been used routinely by the National Nutrition Monitoring Bureau (NNMB) for national level survey in India [19]. The food and nutrients intake of households was compiled, calculated and analysed using the 24- h dietary recall data. Based on the 24-h dietary recall data, a Household Dietary Diversification score (HDDS) and nutrient adequacy ratio (NAR) for each HH was calculated, using standard methods [20-22].

The sampled snacks included food items that were consumed by the HHs during the recall period but did not have fatty acid profile in the Indian food database. Such items were analysed for their fat content and the fatty acid profile. This data was then utilized for the nutrient intake analysis purposes. The snacks sampled included freshly prepared food items or items sold in unlabelled packages. Snacks being sold in standard packaging (i.e. branded snacks from multinational companies) and with food labels were not sampled.

The fat extraction and estimation was done based on the AOAC 996.06 [23] protocol. Confirmation of presence of trans fatty acids (TFA) was done by analysis on a gas chromatograph (GC) equipped with a flame ionization (FID) detector (Nucon Series II, 5700/5765). A fatty acid methyl esters (FAME) mix (GLC-607) and individual fatty acid esters from NuChek Prep Inc. USA, were used to characterize and identify individual fatty acids in the oils extracted from the snacks. All samples were analyzed in duplicates.

### Lipid profile

Blood samples for lipid analysis were taken from a subset of 130 fasting individuals (50 % of HHs surveyed; adult active member between 25–60 years of age). The blood samples were collected between 7.30–9.00 am, by a laboratory technician assisted by a field investigator. Standard procedures were followed for sample collection [24]. The blood lipid profile parameters analysed included total cholesterol, triglycerides, HDL and LDL and were done using recommended kits on a Cobas C311 Auto-analyzer (Roche Diagnostics).

### Anthropometric assessment

Anthropometric measurements (weight and height) were taken on the same individuals from whom the blood sample was collected using standard procedures [25]. The body mass index (BMI) was calculated and classified according to Asian cut-offs [26] for BMI categories i.e. chronic energy deficiency (CED; BMI < 18.5 kg/m^2^), normal (BMI 18.5–22.9 kg/m^2^), overweight (BMI 23.0–27.5 kg/m^2^) and obesity (BMI ≥ 27.5 kg/m^2^).

#### Data analysis

The dietary consumption data was collected at the household level and was used to compute the average daily consumption of various food groups (i.e. standard food groups as defined by ICMR) and nutrients by the household members. These intakes were expressed as per consumption unit (CU) per day, developed by ICMR for different age groups, sex and physical activity level [19, 27]. One consumption unit is defined as the calorie consumption of an average adult man, weighing 60 kg, doing sedentary type of work. The other coefficients are worked out on the basis of calorie requirement proportionately. The different consumption units according to age and sex are given in Table 1. The total consumption units of each household were calculated for all the members present in the household according to different age groups, sex and physical activity level [28]. The nutrient and food intakes were then calculated as per CU as follows.

$$\text{Intake of each food group or nutrient/CU per day}=\frac{\text{Raw amount of each food group or nutrient}}{\text{No. of consumption units}}$$

These intakes were compared with recommended dietary intakes (RDI) and recommended dietary allowances (RDA) for Indians [27, 29].

Intake assessment and calculations were done using the software “DietSoft” (version 1.2.0; 2008–2009; Department of Dietetics; AIIMS & Invincible IDeAS Co., India) which is based on the values in the Nutritive Value of Indian foods database [30] and compared with the recommendations for a sedentary adult male (considered as 1 CU). Previous studies on household food consumption methods that have expressed intake as Adult Consumption Equivalents (ACE; also called Adult Male Equivalent (AME)) have compared the method with individual 24- h dietary recall. The two methods i.e. household and individual dietary recall have shown comparable results for adult individuals [31, 32].

Nutrient Adequacy Ratio (NAR; defined as ratio of subject’s intake of the day to the RDA of that nutrient) is a tool to assess the diet quality and was computed for all nutrients. The NAR was calculated by comparing nutrient intake of study households (expressed as per CU per day) with national recommendations [29] to estimate the nutrient adequacy. The subjects were categorized as those having an adequate (≥1.00), fairly adequate (0.66- <1.00) or inadequate (<0.66) NAR for various nutrients. The energy intake data was expressed as percent of RDA as NAR is not considered a good indicator for assessing adequacy or inadequacy of energy intake [22].

Data was analyzed using R software (version 2.15.0). As the intake of the nutrients was not normally distributed, the average intake over the two days reported in the dietary recall was used to compute the medians and inter- quartile ranges for the food and nutrients. The two days dietary intake measurement was regarded as an estimate of the household’s true intake, subject to random variation due to within and between -HH variations. Coefficients of variation for nutrients for both within- and between- HH were computed. Variance ratios were also estimated. A variance ratio of more than 1 indicated a greater within- than between- HH variance.

In order to correct for measurement error and obtain a better estimate of between- HH variability, we employed a linear model with random intercepts for each household in a repeated measures framework. This takes into account the potential correlations between nutrient intake measurements from same households.

To obtain the estimated probabilities of NAR categories for each nutrient, we assumed a normal distribution for the NARs with mean and variance derived from the overall means and the estimated variance of the random intercept (from the above model). In Table 2, the percentage of HHs (i.e. 100* estimated probabilities) of each NAR category for various nutrients has been shown using both simple proportions and the corrected model. In cases where the distribution of the nutrient intakes was non- normal, we explored transformations (Box- Cox and log transformations; [33]) on the data, with little (in 3 out of 9 nutrients) or no (6 out of 9 nutrients) improvements in mitigating non- normality.

Non- parametric Spearman correlation coefficient was used to investigate the relation between HDDS and NAR of each nutrient. To evaluate the relationship between lipid profile indicators and dietary intake, multiple linear regression was used. Log-transformed variables were used for data that were not normally distributed. The characteristic analysis between those households which participated in lipid profile analysis to those which did not was also analysed using Chi square test for categorical variables and Wilcoxon rank sum test for age. This has been given as a Additional file 1: Table S1.

AIMIL software was used for editing the chromatograms and for calculating the saturated, monounsaturated and polyunsaturated (including TFA content) fatty acid content of the fats extracted from the sampled snacks. The content of each fatty acid in the sample was expressed as a percentage of the total fatty acids in that sample.

### Ethical approval

Ethics approval was obtained from the Public Health Foundation of India’s Institutional Ethics Committee. All procedures involving human subjects were in accordance with the ethical standards of the committee. Written informed consent was obtained from all subjects prior to initiation of the study.

## Results

A total of 261 HHs participated in the study. The average family size was 6 with a “sex ratio” (ratio of males to females) of 1.13. Approximately 46.7 % (*n* = 693/1484) of the study population was in the age group of 19–50 years while 46 % were children (<12 years) and adolescents (12–18 years). The HHs reported an average of 2 working members. Most respondents had received some level of school based education (i.e. any formal schooling of primary or senior secondary level) in 42.5 % of the HHs (Table 2). Eighty-three percent of the residents were native to the slum (i.e. >10 years).

### Dietary intake patterns

The households in the study community were predominantly non- vegetarian (216, 83 %) and majority of the adults (82 %) and children (60 %) consumed two main meals a day. The median monthly food expenditure was INR 5250 (550–16,000); 11 % of the monthly food expenditure was on snacks (median INR 600 [0–5000]). About 85 % of working members reported carrying home cooked meals to their respective workplaces. Those not carrying home cooked meals (15 %) reported eating food purchased from *dhabas* (local eateries; 6.1 %) and food outlets like office canteen, restaurants and food vendors.

### Fat consumption and snacking pattern

As a part of the HH survey, we specifically assessed the fat consumption and snacking pattern of the study HHs.

Cooking medium: Most respondents (15.7 %, *n* = 41) reported using a combination of some refined vegetable oil along with *desi ghee* (*clarified butter*)/butter as a cooking medium daily. About 56 % of HHs reported using 2 or more cooking mediums. The most common cooking medium was refined vegetable oil (with mustard and soybean being the commonest) in the HHs. The consumption of *vanaspati* (the most common source of PHVO in Indian diet) was reported by 15 % of HHs (*n* = 38), in addition to the other cooking mediums being used in the HHs. The reported median consumption of *vanaspati* in the HHs using it was 24 g/CU/day (4–131 g/CU/day).

**Fig. 2:**
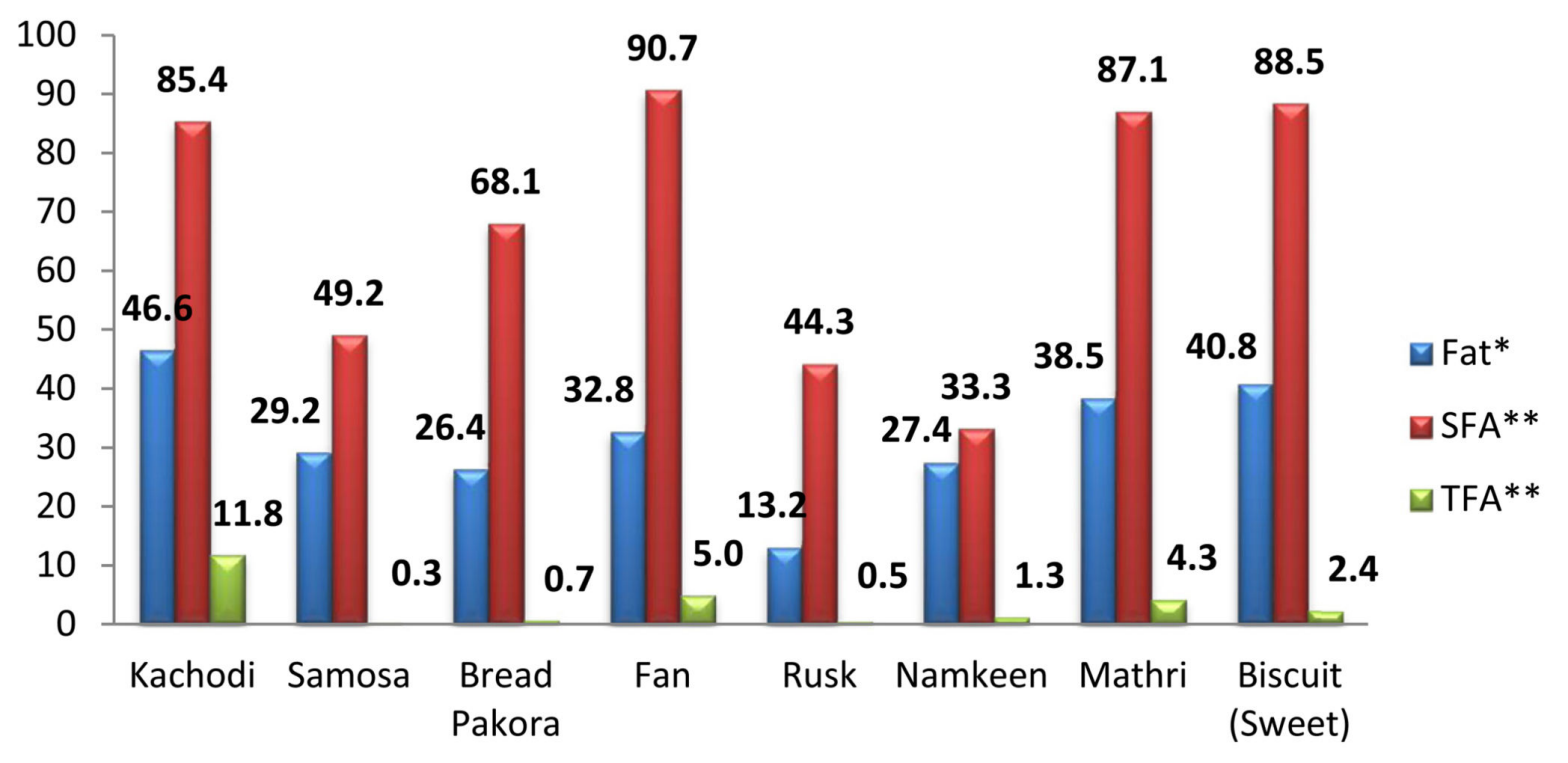
Fat, Saturated fatty acids (SFA) and Trans fatty acids (TFA) content of commonly consumed snacks

Snacking pattern: The community consumed snacks that were either homemade or purchased from the market. The market snacks were either packaged or freshly prepared. The majority (96 %) of the families reported consuming some kind of snack from the above mentioned categories. Only 18.4 % (48) HHs reported consumption of the home- made snacks (*pakora*, *cheela*, *paranthas*, *halwa*, *seviyaan*). The deep- fried snacks were consumed by 8.4 % HHs with majority consuming them on a fortnightly or monthly basis (3 %). About 14.2 % of HHs reported consumption of shallow- fried snacks with majority reporting a weekly consumption frequency (6.1 %). About 66 % HHs reported consuming packaged (both branded and unlabelled) snacks; and a two- third of these reported consuming them daily. A list of the most commonly consumed snacks is included as Additional file 2: Table S2. Consumption of freshly prepared commercially available snacks like *samosa*, *kachodi*, other savouries and traditional sweets like *laddoo*, *soan papdi*, etc. was observed in 38 % of HHs with consumption on a weekly (10 %) or fortnightly (10 %) basis. Majority (43 %) reported that the freshly prepared snacks were fried and among them *samosa* (30 %) was the most common. The fat content of freshly prepared and unlabelled packaged snacks from the market were assessed as part of the present study. The total fat content of these snacks ranged from 0.6–26 g in usual servings. Most of the snacks (24/29) sampled and analysed were found to contain TFA ranging from 0.26–22.96 % of total fatty acids. The fat, SFA and TFA content assessed in these snacks are shown in Fig. 2. The MUFA and PUFA content of the snacks expressed as % of total fatty acids ranged from 1.81 % (in *kachodi*) to 41.56 % (in *samosa*) and 0.08 % (in biscuit) to 55.27 % (in *namkeen*) respectively.

The median fat consumed from snacks per CU per day was 12.3 g (0.3–90.2 g, *n* = 236). This was in addition to the consumption from regular visible fat (i.e. fat added during cooking) from HH cooking mediums. Approximately 23 % (60) HHs were consuming more than 10 % of daily energy from fats present in the commercially prepared snacks. Consumption of commercially prepared snacks was the major source of trans fats in the diets of the study community. The median trans fat consumption from these snacks was 0.67 g/ CU/ day (range: 0.01–11.44 g/ CU/ day). The majority of the HHs (87 %; *n* = 288) showed a consumption of less than 1 % of daily energy while about 13 % (33) HHs were consuming more than 1 % daily energy from TFA from commercially prepared snacks.

### Food and nutrient intakes

The median consumption of various food groups as assessed from the dietary recall data (as g/CU/day) is given in Table 3. Among various food groups, a higher consumption of visible fats with a median intake of 37 g/CU/day (5–152 g/CU/day) was reported. Median milk intake of 117 ml/CU/day (7–654 ml/CU/day) was less than 50 % of the RDI (300 ml). The median intake of vegetables including roots and tubers, green leafy and other vegetables was one- fourth (130 g/CU/day [0–442 g/CU/day]) of the RDI (500 g) and predominantly included roots and tubers. The intake of green leafy and other vegetables was found to be less than 25 % of the recommended value of 100g and 200 g respectively. The median meat consumption was found to be more than the RDI (41.7 g/CU/day [0.3–260.8 g/CU/day]).

The median nutrient intakes and nutrient adequacy (as %RDA) of various nutrients are shown in Table 4. Median energy (1829 kcal, 79 % of RDA) and fat intake (52.5 g,^1^ 102 % RDA) was adequate. Vitamin A adequacy was only 15 % of RDA in the study population.

The within- HH variation was found to be more than between- HH variation for vitamin C, thiamine, niacin, folic acid, iron and protein (Additional file 3: Table S3). The variance ratio for various nutrients ranged from 0.50 (calcium) to 3.46 (vitamin C). In more than 85 % of the HHs, vitamin A and riboflavin intake was found to be inadequate (<0.66 NAR) which didn’t change whereas the percentage of HHs with inadequate intake of folate and calcium decreased, after correcting using repeated measures random intercepts model (Table 4).

### Household dietary diversification score (HDDS)

Based on the food consumed in the past 24 h, the HDDS ranged from 6 to 11 with a mean (±SD) score of 7.87 (1.07). All HHs had consumed cereals (wheat flour or rice), sweets (sugar), oils & fats (vegetable oil) and spices, beverages & condiments (salt) in the past 2 days. The most commonly consumed beverage was tea. About a quarter of the HHs (26 %) consumed dark GLVs whereas only 12 % consumed vitamin A rich vegetables & tubers. Vitamin A rich fruits were rarely consumed (6 % HHs). Milk consumption was observed in 45 % of HHs. The correlation between HDDS and nutrient adequacy expressed as NAR for different nutrients are shown in Table 5. The correlation ranged from 0.031 for niacin to 0.322 for calcium. The correlation coefficients for protein (*r* = 0.282, *p* = 0.000), vitamin A (*r* = 0.307, *p* = 0.000), riboflavin (*r* = 0.216, *p* = 0.000) and calcium (*r* = 0.322, *p* = 0.000) were found to be significant.

### Nutritional status

Nutritional status was assessed in 132 individuals (69 % females). The study participants showed a mean (SD) BMI of 25.2 ± 4.8 kg/m^2^ with a higher BMI in females compared to males (25.7 ± 4.8 kg/m^2^ vs. 24.0 ± 4.5 kg/m^2^). The prevalence of CED (BMI < 18.5 kg/m^2^) was about 2.3 % in males and 5.3 % in females. More than half of the study participants (66.7 %) were overweight/obese (≥23.0 kg/m^2^) with overweight observed (23.0–27.5 kg/m^2^) in 45.5 % participants and obesity (≥27.5 kg/m^2^) in 21.2 % participants. The prevalence of both overweight and obesity was higher in females (34.1 % overweight; 16.6 % obesity) as compared to males (11.4 % over-weight; 4.5 % obesity).

The estimated mean cholesterol levels were (180.6 ± 31.1 mg/dl in males and 177.1 ± 36.8 mg/dl in females) which are below the recommended cut- offs [34]. The triglyceride levels were higher in males (150.2 ± 66.1 mg/dl) than females (125.3 ± 49.9 mg/dl) when compared with the recommended levels. The mean HDL-C was normal as per cut- offs recommended for males (42.94 ± 13.8 mg/dl) but was lower than recommended levels for females (45.0 ± 11.3 mg/dl). The mean LDL-C (84.4 ± 22.5 mg/dl males; 88.2 ± 30.0 mg/dl females) was within recommended levels.

A significant proportion of the subjects had a dyslipidemic profile with low HDL (46.3 % & 69.2 % of males & females respectively) and hypertriglyceridemia (in 39.0 % of males and 25.3 % of females) being most common. About 25 % of both males and females had hypercholesterolemia (>200 mg/dl) while 3.3 % of females showed higher LDL-C levels than cut- offs (<159 mg/dl). Analysis of lipid profiles for correlations with dietary intake did not show any significant correlations.

## Discussion

The purpose of the study was to assess the dietary intake patterns and diversity with a specific focus on fat intake and snacking pattern in an urban slum in North India. Some important observations were made regarding the dietary consumption patterns.

With regard to fat consumption, a majority of the population used a combination of cooking mediums that included refined vegetable oils along with sources of saturated fats. The consumption of PHVOs (predominant source of TFA) as a household cooking medium was found to be low (15 % of HHs). Other studies have however reported varying levels of PHVO intake across regions in India, with a higher intake of en% from TFA in urban slum residents [35, 36]. A quarter of HHs were consuming >10 % of daily energy from fats present in snacks. Amongst a total of 29 snacks sampled and analysed, 24 samples were high in fat content (with a median saturated fat content of 61.7 % of total fatty acids) and contained trans fats, albeit in varying concentrations. The consumption trend seen has implications for dietary quality as these snacks are known to be energy dense with low micronutrient content [37]. However, despite the consumption of these snacks, the energy consumption from TFA in majority of the HHs was <1 % which is in concordance with the WHO recommendations [15]. No study in India, to the best of our knowledge has reported the trans fatty acid intake pattern in the low SES population residing in urban areas. The consumption patterns observed with regards to commercially prepared snacks reflects the changing food preferences and the influence of marketing across diverse community settings [38, 39]. The regular consumption of nutrient poor and energy dense snacks aggravates an already compromised health status created by low intakes of fresh fruits and vegetables as reported in the present study. This is likely to have potentially adverse implications for future chronic disease development particularly in a resource poor population [40, 41].

Our findings on intakes of different food groups are consistent with previous studies (e.g. in Mumbai slums and in urban North and South India) showing an inadequate intake of pulses, roots and tubers, other vegetables, GLVs and fruits but a higher intake than recommended of fats and oils, milk and sugars in adults [42, 43]. We also observed an adequate median consumption of cereals, meats, fats and sugars but a low intake of pulses and milk along with very low intakes of vegetables, roots and tubers, GLVs and fruits. The calculated HDDS (mean: 7.87) also reflected the inadequate consumption of dark GLVs, vitamin A rich fruits, vegetables and tubers. Notably, none of the participants met the recommended intake guidelines for vegetables of 500gm /day. A high median meat and meat products consumption in the study community perhaps indicates the effect of nutrition transition on food consumption pattern. Though these are sources of protein in the diet, they may contribute to high saturated fat intake and increase the risk of non- communicable diseases [44, 45].

The macronutrient intake was adequate but micronutrient intakes were generally low. Amongst these, more than two- thirds of the study population had inadequate (<0.66 NAR) vitamin A (98.6 % HHs) and riboflavin (87.5 % HHs) intake while almost half of the population showed inadequate folate (51 % HHs) intake. The recent NSSO [17] surveys also indicate a change in the food consumption in urban areas over a period 1993–2009 with a reduction in intake of staples (cereals and pulses) and an increase in intake of more energy- dense foods, particularly fats. The NARs for most nutrients (except for vitamin C, thiamine, niacin and iron) correlated significantly with HDDS. Other studies have also shown inadequate intake of folate, vitamin A and iron in adults and young adolescents in urban and rural north India [22, 46].

A high prevalence of overweight/obesity (i.e. more than 50 % of participants) was observed in the urban population surveyed. This could be due to a sedentary lifestyle which is partly because of the community structures that are typical of such congested urban conglomerations [47]. Prevalence of dyslipidemia (prevalence of overall hypertriglyceridemia in 29.5 % and hypercholesterolemia in 25.8 % of participants) was seen along with an adequate fat intake. An association between dyslipidemia with decreased physical activity and increased fat intakes has also been reported in some previous studies in similar settings [48, 49].

## Conclusions

The key findings of the present study highlight the poor dietary diversity with a predominantly cereals and fats based diet and a high prevalence of overweight. An overall contribution of commercially prepared food towards fat intake of poor quality is also a cause of concern. This underscores the definite impact of urbanization and nutrition transition across diverse communities [50]. To the best of our knowledge, our study is the first to have reported data on trans fat intake, HDDS and NAR in urban low SES communities in India.

Policies should encompass strategies that raise awareness on healthy diet and lifestyle practices with emphasis on quantity and quality of nutrient intake. This is even more imperative in communities that may have low awareness of disease implications and limited resources for and access to healthier options.

### Study limitations

While the design of our study was appropriate for making conclusions regarding the immediate study community and its neighbouring areas, we do admit that the vast geographical expanse and cultural diversity of India cannot be captured in one study. Hence, while some of the results regarding the need for policy and program measures may be generalizable, the contextual nature of dietary practices and results gleaned thereof cannot be stated to be representative of nationwide consumption patterns. The dietary intake in our study was assessed at household level and not at individual level. The intakes are thus reported at “per consumption unit” level. Due to logistic feasibility, the 2 day 24- h dietary recall may or may not have captured dietary intake for both a weekdays and weekends for all the HHs. As the nutrients intake data was non- normal, there could be an overestimation of the proportion above/below the thresholds, in particular for the nutrients with high variance ratios. Further, while a single person was most often responsible for managing the kitchen duties such as purchasing, cooking and handling the kitchen inventory, there would be some limitations in the ability of a single person to reliably report household- level consumption. Though we took the most reliable person to report the household dietary intake but didn’t verify how this might have affected the reporting of the household intake. The household 24- h dietary recall was not validated in the present study.

## Supplementary Material

Additional table 1

Additional table 2

Additional table 3

Additional table 4

## Figures and Tables

**Table 1:** Consumption units^a^ according to age and sex developed by Indian Council of Medical Research^b^

Category	Consumption Unit
Adult male (Sedentary worker)	1.0
Adult male (Moderate worker)	1.2
Adult male (Heavy worker)	1.6
Adult female (Sedentary)	0.8
Adult female (Moderate)	0.9
Adult female (Heavy)	1.2
Adolescent (12–21 years)	1.0
Children (9–12 years)	0.8
Children (7–9 years)	0.7
Children (5–7 years)	0.6
Children (3–5 years)	0.5
Children (1–3 years)	0.4

a Consumption Units (CU): One consumption unit is defined as the calorie consumption of an average adult man, weighing 60 kg, doing sedentary type of work. The other coefficients are worked out on the basis of calorie requirement proportionately. Indian Council of Medical Research (ICMR) developed these coefficients for different age groups, sex and physical activity

b Reference 28: Nutrition Surveys in India; Ministry of Women and Child Development; http://wcd.nic.in/research/nti1947/7.1%20Nutrition%20Surveys%20in%20India%20pr%203.2.pdf

**Table 2:** Socio- demographic profile of the study households (*N* = 261)

Socio- demographic Characteristic	Urban Slums [n (%)
*Gender of respondent:*	
Female	233 (89.3)
Male	28 (10.7)
*Educational status of respondent*	
Illiterate	77 (29.6)
Can read and write with no formal education	70 (26.8)
Formal Schooling	111 (42.5)
Formal College	3 (1.1)
*Religion:*	
Hindu	131 (50.2)
Muslim	130 (49.8)
*Occupation of Head Of Household*	
Business^a^	69 (26.5)
Self- employed^b^	98 (37.6)
Non- government job^c^	21 (8.0)
Government job	10 (3.8)
Non working/ Retired^d^	63 (24.1)
*Monthly Income (NR)*[51]	
<3000	10 (3.8)
3000–10,000	204 (78.2)
>10,000	47 (18.0)

a Business includes shop- keeper and involved in small business;

b Self- employed includes artisans, involved in religious activities;

c Non- government job includes those working in a private firm/ organization;

d Non- working includes unemployed, retired and housewife

**Table 3:** Median food groups intake (expressed as per consumption unit) in study households (*N* = 261)

Food group	Indian Council of Medical Research^a^ (2010) Recommended intakes	Intake/ Consumption unit^b^[Median (Range)]
Cereals (g)	375	296.5 (73.8–916.7)
Fats (g)	25	36.9 (5.1–151.7)
Milk (g)	300	117.1 (7.5–653.7)
Sugars (g)	20	20.0 (1.4–137.9)
Fruits (g)	100	34.3 (0.7–219.0)
Roots & tubers (g)^c^	200	95.3 (3.1–373.9) (*n* = 257)
Green Leafy Vegetables (g)^c,d^	100	**15.2 (0.8–166.7)** (*n* = 94)
Other Vegetables (g)^c,d^	200	**41.3 (0.8–245.0)** (*n* = 128)
Total Vegetables (g)	500	130.0 (0–441.8)
Meat (g)^c^	30	41.7 (0.3–260.8) (*n* = 109)
Pulses (g)^c^	45	29.6 (0.9–206.4) (*n* = 238)

a Indian Council of Medical Research (ICMR): Dietary Guidelines for Indians-A manual. New Delhi: National Institute of Nutrition, Indian Council of Medical Research; 2010

b Consumption Units (CU); One consumption unit is defined as the calorie consumption of an average adult man, weighing 60 kg, doing sedentary type of work. The other coefficients are worked out on the basis of calorie requirement proportionately. Indian Council of Medical Research (ICMR) developed these coefficients for different age groups, sex and physical activity

c Median intake of meat, pulses and kinds of vegetables given with number of HHs consuming [Median (Inter- Quartile Range (n))]

d Intakes are less than 25 % of the recommendations; values indicated in bold

**Table 4:** Median intake with adequacy of nutrients (expressed as per consumption unit^a^) and percentage distribution of Nutrient Adequacy Ratio in study households (*N* = 261)

Nutrient	Recommended Dietary Allowances^b^ (2010)	Median Intake	Adequacy (% RDA^c^)	Nutrient Adequacy ratio (% of Households)					
				Inadequate		Fairly adequate		Adequate	
				(<0.66)		(0.66- <1.00)		(≥1.00)	
				Original	Corrected^d^	Original	Corrected^d^	Original	Corrected^d^
Protein^e^ (g)	60	49.8	83	22.6	26.3	51.3	42.5	26.1	31.2
Vitamin A (μg)	600	88.5	15	**92.0** ^f^	**98.6** ^f^	6.9	1.4	1.1	0.0
Vitamin C^e^ (mg)	40	35.7	89	37.2	40.2	19.2	18.2	43.7	41.6
Thiamine^e^ (mg)	1.2	1.3	107	10.7	16.1	31.8	27.5	57.5	56.4
Riboflavin (mg)	1.4	0.6	42	**86.6** ^f^	**87.5** ^f^	12.6	12.4	0.8	0.1
Niacin (mg)	16	11.8	74	37.5	35.1	39.5	39.3	23.0	25.6
Calcium (mg)	600	383.9	64	**52.9** ^f^	40.8^f^	31.4	43.4	15.7	15.8
Folate (μg)	200	120.7	60	**58.2** ^f^	**51.4** ^f^	29.9	37.6	11.9	11.0
Iron (mg)	17	13.0	76	37.2	33.2	35.6	35.4	27.2	31.4

a Consumption Units (CU): One consumption unit is defined as the calorie consumption of an average adult man, weighing 60 kg, doing sedentary type of work. The other coefficients are worked out on the basis of calorie requirement proportionately. Indian Council of Medical Research (ICMR) developed these coefficients for different age groups, sex and physical activity

b Indian Council of Medical Research: A report of the expert group of Indian Council of Medical Research; Nutrient Requirements and Recommended Dietary Allowances for Indians, 2010

c RDA: Recommended Dietary Allowances

d Percentage calculations after correction were obtained from a normal distribution with mean and variance derived from the overall means and estimated between- household variability, from repeated measures random intercepts model

e For protein, vitamin C and thiamine, probabilities were based on calculations with transformed data and details are provided in Additional file 4: Table S4

f Values in bold show the inadequacy (<0.66 Nutrient Adequacy Ratio) in more than 50 % of the households

**Table 5:** Spearman’s Correlation coefficient between Household Dietary Diversification Score^a^ and Nutrient Adequacy Ratio

Nutrients	Median Nutrient Adequacy Ratio	Spearman Correlation Coefficient	*p* value
Protein (g/ CU^b^/ day)	0.83	0.282	0.000*
Vitamin A (μg/ CU/ day)	0.15	0.307	0.000*
Vitamin C (mg/ CU/ day)	0.89	0.079	0.202
Thiamine (mg/ CU/ day)	1.07	0.051	0.414
Riboflavin (mg/ CU/ day)	0.42	0.216	0.000*
Niacin (mg/ CU/ day)	0.74	0.031	0.619
Calcium (mg/ CU/ day)	0.64	0.322	0.000*
Folate (μg/ CU/ day)	0.60	0.144	0.020
Iron (mg/ CU/ day)	0.76	0.053	0.397

a Median Household Dietary Diversification Score (HDDS) of 8 used for correlation calculations

b Consumption Units (CU): One consumption unit is defined as the calorie consumption of an average adult man, weighing 60 kg, doing sedentary type of work. The other coefficients are worked out on the basis of calorie requirement proportionately. Indian Council of Medical Research (ICMR) developed these coefficients for different age groups, sex and physical activity

* Significant at 0.01 level

## Footnotes

1 Recommendation computed as energy contribution by fat (25 % en) i.e. 51 g

## Abbreviations

HDDS: Household Dietary Diversification Score
PHVOs: Partially Hydrogenated Vegetable oils
HDL-C: High Density Lipoprotein Cholesterol
NFHS: National Family Health Survey
NCDs: Non-Communicable Diseases
CVD: Cardio-Vascular Disease
NMMB: National Nutrition Monitoring Bureau
NSSO: National Sample Survey Organization
SES: Socio-Economic Status
HHs: Households
NAR: Nutrient Adequacy Ratio
AOAC: Association of Official Analytical Chemists
FID: Flame Ionization Detector
FAMEs: Fatty Acid Methyl Esters
GC: Gas Chromatography
LDL-C: Low Density Lipoprotein Cholesterol
BMI: Body Mass Index
CED: Chronic Energy Deficiency
CU: Consumption Unit
RDI: Recommended Dietary Intakes
RDA: Recommended Dietary Allowances
ICMR: Indian Council of Medical Research
GLVs: Green Leafy Vegetables
ACE: Adult Consumption Equivalent
AME: Adult Male Equivalent
SFA: Saturated Fatty Acids
TFA: Trans Fatty Acids
MUFA: Mono-Unsaturated Fatty Acids
PUFA: Poly-Unsaturated Fatty Acids
WHO: World Health Organization
