# Supplementary material for: Quantitative estimates of dietary intake with special emphasis on snacking pattern and nutritional status of free living adults in urban slums of Delhi: impact of nutrition transition
Source: BMC Nutr. Author manuscript; Available in PMC 2016 Feb 23. (PMC4763040; doi:10.1186/s40795-015-0018-6)
Supplement: Additional table 1 [file NIHMS67114-supplement-Additional_table_1.pdf]

Supplementary Table 1 Characteristic analysis between those households which participated in lipid profile analysis and those which did not

Characteristics	Without Lipid Profile (n= 131)	With Lipid Profile (n=130)	p value*
Median Age (IQR)	35 (26-45)	36.5 (30-44)	0.1736
Gender (%)			
Male	11 (8.40)	18 (13.85)	0.161
Female	120 (91.60)	112 (86.15)	
Wealth (%)			
<3000	5 (3.82)	5 (3.85)	0.830
3000- 10000	100 (76.34)	103 (79.23)	
>10000	26 (19.85)	22 (16.92)	
Education status (%)			
Illiterate	39 (29.77)	37 (28.46)	0.924
Formal Schooling	55 (41.98)	57 (43.85)	
Formal College	1 (0.76)	2 (1.54)	
Can read and write with no formal education	36 (27.48)	34 (26.15)	

*p value below 0.01 is significant

The characteristic analysis clearly showed that the households which participated in the lipid profile and anthropometric measures were not significantly different from those which did not participate. No difference in the socio- demographic profile (in terms of age, gender, wealth and educational status) was found between the households.
