# Supplementary material for: Quantitative estimates of dietary intake with special emphasis on snacking pattern and nutritional status of free living adults in urban slums of Delhi: impact of nutrition transition
Source: BMC Nutr. Author manuscript; Available in PMC 2016 Feb 23. (PMC4763040; doi:10.1186/s40795-015-0018-6)
Supplement: Additional table 2 [file NIHMS67114-supplement-Additional_table_2.pdf]

Supplementary Table 2 Description of Snacks

Namkeens- salted tidbits in a pack- mix of wheat and lentils

Rusk- dry biscuit, twice baked bread

Fan- wafer shaped flaky salted pastry puffs

Samosa- potato stuffed deep fried salted refined wheat flour pockets

Kachodi- deep fried salted puffed bread made of wheat flour

Ladoo- ball shaped sweet made of fried gramflour granules dipped in sugar

Soan papdi- flaky sweet made of gramflour

Pakora- deep fried snacks of vegetables like potato, cauliflower, onion etc.

Cheela- a pancake made of wheat flour or gram flour

Paranthas- a flatbread made of wheat flour, prepared by pan frying

Halwa- a kind of dessert made of flour, butter, sugar or nuts

Seviyaan- roasted thin vermicelli, a kind of pasta
