# Supplementary material for: Quantitative estimates of dietary intake with special emphasis on snacking pattern and nutritional status of free living adults in urban slums of Delhi: impact of nutrition transition
Source: BMC Nutr. Author manuscript; Available in PMC 2016 Feb 23. (PMC4763040; doi:10.1186/s40795-015-0018-6)
Supplement: Additional table 3 [file NIHMS67114-supplement-Additional_table_3.pdf]

Supplementary Table 3 Coefficients of variation and variance ratio for different nutrients per consumption unit

**Nutrient**	**Coefficient of variation (%)**		**Variance Ratio (S_w^2/S_b^2)**
	**Within- Household (CV_w)**	**Between- Household (CV_b)**	
Energy	23.68	28.08	0.71
Protein	**33.80**	27.34	**1.53**
Fat	36.37	45.44	0.64
Vitamin A	67.05	91.47	0.54
Vitamin C	**92.22**	49.56	**3.46**
Thiamine	**36.61**	33.46	**1.20**
Riboflavin	31.78	32.82	0.94
Niacin	**33.62**	32.88	**1.05**
Calcium	30.17	42.61	0.50
Folic Acid	**40.41**	31.33	**1.66**
Iron	**37.00**	32.98	**1.26**

Values in bold indicate greater within- household than between- household coefficient of variation for nutrients
