# Supplementary material for: Quantitative estimates of dietary intake with special emphasis on snacking pattern and nutritional status of free living adults in urban slums of Delhi: impact of nutrition transition
Source: BMC Nutr. Author manuscript; Available in PMC 2016 Feb 23. (PMC4763040; doi:10.1186/s40795-015-0018-6)
Supplement: Additional table 4 [file NIHMS67114-supplement-Additional_table_4.pdf]

Supplementary Table 4: Percentage of households[*] for different Nutrient Adequacy Ratio categories in study households

**Nutrient**	**Lambda**[**]	**Mean**	**Standard Deviation**[ǂ]	**Nutrient Adequacy Ratio**[ǂǂ] **(% of Households)**		
				**<0.66**	**0.66- <1.0**	**≥1.0**
Protein	1	0.866	0.320	26.0	40.2	33.8
	0.75	-0.155	0.317	26.3	42.5	31.2
Vitamin A	1	0.233	0.194	98.6	1.4	0.0
Vitamin C	1	1.046	0.956	34.3	13.7	52.0
	0.5	-0.173	0.815	40.2	18.2	41.6
Calcium	1	0.723	0.274	40.8	43.4	15.8
Thiamine	1	1.145	0.513	17.2	21.6	61.2
	0.5	0.073	0.452	16.1	27.5	56.4
Riboflavin	1	0.451	0.181	87.5	12.4	0.1
Niacin	1	0.785	0.328	35.1	39.3	25.6
Iron	1	0.820	0.369	33.2	35.4	31.4
Folate	1	0.650	0.285	51.4	37.6	11.0

[*] Percentage of households= 100* estimated probabilities;

[**]Lambda is the tuning parameter in the Box- Cox transformation. Value of the parameter lambda equals 1 corresponds to the original data, while any other value corresponds to the transformed response variable (y^ lambda – 1)/ lambda. Results for transformed data are only shown in cases where it provided better results than the original data;

[ǂ] Standard deviation is estimated from repeated measures random intercepts model using both the measurements for each individual household;

[ǂǂ] The probabilities for each Nutrient Adequacy Ratio (NAR) category are computed using normal distributions with the mean and standard deviations from the repeated measures random intercepts model
